# Multi-phenotype analysis for enhanced classification of 11 herpes simplex virus 1 strains

**DOI:** 10.1099/jgv.0.001780

**Published:** 2022-10-19

**Authors:** Sarah N. Dweikat, Daniel W. Renner, Christopher D. Bowen, Moriah L. Szpara

**Affiliations:** ^1^​ Department of Biology, University Park, USA; ^2^​ Center for Infectious Disease Dynamics, Huck Institutes of the Life Sciences, USA; ^3^​ Department of Biochemistry and Molecular Biology, The Pennsylvania State University, University Park, USA

**Keywords:** comparative genomics, human herpesvirus 1, plaque phenotype, viral diversity

## Abstract

Herpes simplex virus 1 (HSV1) is best known for causing oral lesions and mild clinical symptoms, but it can produce a significant range of disease severities and rates of reactivation. To better understand this phenotypic variation, we characterized 11 HSV1 strains that were isolated from individuals with diverse infection outcomes. We provide new data on genomic and *in vitro* plaque phenotype analysis for these isolates and compare these data to previously reported quantitation of the disease phenotype of each strain in a murine animal model. We show that integration of these three types of data permitted clustering of these HSV1 strains into four groups that were not distinguishable by any single dataset alone, highlighting the benefits of combinatorial multi-parameter phenotyping. Two strains (group 1) produced a partially or largely syncytial plaque phenotype and attenuated disease phenotypes in mice. Three strains of intermediate plaque size, causing severe disease in mice, were genetically clustered to a second group (group 2). Six strains with the smallest average plaque sizes were separated into two subgroups (groups 3 and 4) based on their different genetic clustering and disease severity in mice. Comparative genomics and network graph analysis suggested a separation of HSV1 isolates with attenuated vs. virulent phenotypes. These observations imply that virulence phenotypes of these strains may be traceable to genetic variation within the HSV1 population.

## Introduction

Herpes simplex virus 1 (HSV1) is a human alphaherpesvirus that infects approximately 70 % of the global population [1]. HSV1 infection is often mild, commonly appearing as recurrent oral or genital lesions, and it is often considered to be clinically tolerable [2]. At a relatively low frequency, however, HSV1 can produce serious disease outcomes in the host, including ocular infections leading to blindness or infections that progress to encephalitis or meningitis [3, 4]. The ability of HSV1 to infect and be tolerated in such a large proportion of the population offers an opportunity to dissect differences in the observed virulence or pathogenicity of different viral isolates [5–8].

Lab-adapted strains typically used in studies of HSV do not necessarily provide an accurate reflection of the natural diversity that occurs across the millions of individuals currently infected with this pathogen [9–12]. It is important to utilize clinical isolates to better represent HSV diversity and its corresponding phenotypes. HSV1 diversity in the laboratory can be assessed based on plaque phenotype in cell culture [13, 14] and by infection assays in animal models, where the time to death and/or rate of mortality at a given viral dose can be used as a measure of virulence [15–17]. Another approach to characterizing the nature of viral variation is to compare whole genomes and within-population variation by deep sequencing [10, 18, 19]. The goal of this study was to integrate these three experimental data types and look for correlations between viral genetics, cell-based plaque phenotypes, and virulence in a murine animal model.

A detailed understanding of the variability and genetic features of HSV1 requires the characterization of numerous viral strains that reflect the breadth of phenotypic outcomes observed in animal models and in humans. A previous report by Dix *et al*. [16] quantified murine disease severity for 23 strains of HSV1, including both clinical isolates and lab-adapted strains. While this study did not include the same endpoints and sex-balance as current animal studies, the sheer number of comparisons it entailed have been an invaluable resource for later studies [6, 20–22]. Results from Dix et al. showed that clinical isolates were generally more virulent than lab-adapted strains, although with a wide range of strain-specific virulence differences within each group [16]. The study also included a dose reduction series for each viral strain to determine the LD_50_ and it compared results from both footpad and intracerebral routes of inoculation. This collection of strains provides a valuable resource for further investigation because of its highly detailed murine phenotype data.

We report the results of a more detailed investigation of 11 clinical isolates of HSV1 from this previous study [16]. The strains were derived from eight individuals, with a range of disease outcomes and severities. We integrate information from plaque phenotype in cells and murine models of infection with whole-genome sequence data to categorize these strains. This multi-phenotype approach allowed us to identify unexpected relationships among these isolates. For example, data showed that an individual with known dual infection could harbour HSV1 strains of distinct cellular phenotype, genotype and murine virulence severity. We also found that two strains collected from different individuals were genetically near-identical, but distinct in their *in vivo* virulence and plaque phenotype. This study supports multi-parameter phenotyping as an effective strategy to link HSV genetic features to disease outcomes.

## Methods

### Viral and cell culture

Viral stocks for each of the 11 samples were grown in MRC-5 cells, a line of normal human lung fibroblast cells (ATCC, CCL-171). Cells were grown in Eagle’s Minimum Essential Media (EMEM) with the addition of 10 % Fetal Bovine Serum, 1× penicillin-streptomycin (Life Technologies—Gibco), 2 mMl-Glutamine (Thermo Fischer Scientific-HyClone), and 0.2 X G-5 supplement (Thermo Fischer Scientific). A master stock was produced using MRC-5 cultures infected at an m.o.i. of 0.01 and harvested when the majority of cells had lysed due to viral infection. The titre of each viral stock was determined by limiting dilution on monolayers of Vero cells, a line of African green monkey kidney cells (ATCC, CCL-81), under methylcellulose. Vero cells were grown in Dulbecco’s Modified Eagle Media (DMEM) 10 % Fetal Bovine Serum, 1× penicillin-streptomycin (Life Technologies—Gibco) and 2 mMl-Glutamine (Thermo Fischer Scientific-HyClone).

### Viral DNA nucleocapsid preparation

In order to obtain enough viral DNA for a nucleocapsid prep, the master stock was used to infect MRC5 cultures at an m.o.i. of 5. The viral DNA was isolated according to the nucleocapsid preparation protocol previously described [23]. Briefly, infected cells were collected 24 h post-infection, and cell pellets were extracted twice using Freon. Viral nucleocapsids were extracted using a glycerol step gradient. The nucleocapsids were lysed using SDS and proteinase K, and DNA was separated out using phenol-chloroform extraction. The viral genomic DNA was then ethanol precipitated and resuspended in TE (10 mM Tris, pH7.6; 1 mM EDTA).

### Plaque characterization

For plaque visualization and characterization, Vero cells were infected at an m.o.i. of 0.01 with each of the 11 strains of HSV1 and grown under a methylcellulose overlay. The cells were fixed and stained with methanol and methylene blue after 72 h. Images were collected using a Nikon Ti Eclipse microscope and NIS-Elements AR version 4.30.02 software. To obtain plaque size, 100 plaques were measured for each strain and individual plaque size was calculated using ImageJ.

### Next-generation sequencing and genome assembly

Genomic DNA from each HSV1 sample was prepared for sequencing on an Illumina MiSeq using the Illumina TruSeq DNA sample prep kit. Viral nucleocapsid DNA was fragmented using a Covaris M220 sonicator with the following parameters: 60 s duration, peak power 50, 10 % duty cycle, at 4 °C. Barcoded Truseq libraries were sequenced on an Illumina MiSeq (v3 chemistry) to obtain 300×300 paired-end reads according to manufacturer instructions. Viral genomes were assembled using the Viral Genome Assembly (VirGA) pipeline [24]. Briefly, the pipeline incorporates multiple quality-control measures from Trimmomatic (58) to remove any adaptor sequence from library preparation, low-quality bases (minimum Phred score 30, 15 bp window size), short-read fragments (minimum size 30 bp), and any unpaired reads. Any sequence reads that map to the host cells used for viral propagation are also removed. SSAKE is then used for *de novo* assembly followed by extension of contigs using Celera and GapFiller. These contigs are then ordered according to the reference genome for HSV1 (strain 17 accession JN555585) and the contigs with the best match are stitched to form a consensus genome using mafnet (a VirGA specific script). Following genome assembly, additional quality-control measures are implemented to identify gaps, flag areas of low coverage, and note any sequence polymorphisms between strains.

### Genetic distance and sequence identity

Genome-wide alignments were prepared for the 11 strains using mafft [25]. This alignment utilized trimmed consensus genomes, leaving out the terminal copies of the long and short repeat regions (TRL/TRS) [24]. These alignments were used to construct phylogenetic networks with the NeighborNet method in SplitsTree4 [26]. Previously published genomes used for comparison are listed in Table S2. Gaps in alignment were excluded in construction of the network graphs to avoid artificial groupings due to misalignment or incomplete sequence assemblies. Uncorrected *P* distances were used to illustrate relationships between viral strains. Individual ORF networks were constructed from ORF sequences aligned using ClustalW2, and the same parameters as used for construction of whole-genome network graphs. Custom Python scripts were used to calculate percent identity and quantify the number of synonymous vs. non-synonymous differences for individual gene and protein alignments. RS1 was excluded from identity analysis due to incomplete sequence assembly in several strains and resulting poor alignment.

## Results

**Fig. 1.**
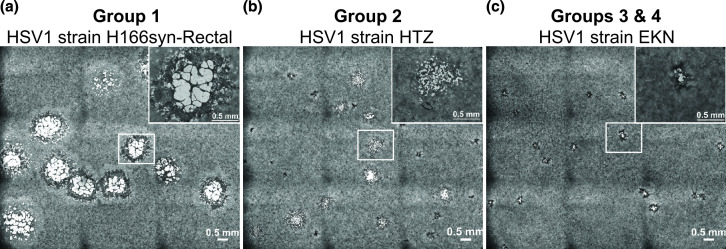
Representative HSV1 plaque images exemplify three categories of plaque phenotypes in cell culture: large or syncytial, intermediate, and small. Vero cells were infected for 72 h with HSV1 strain (a) H166syn-Rectal, (b) HTZ or (c) EKN and stained with methylene blue. Panel (a) represents group 1, with ‘large’ plaques that are partially or fully syncytial. Panel (b) represents group 2 with ‘intermediate’ sized plaques. Panel (c) represents groups 3 and 4, with ‘small’ plaques. The scale bar for each image represents 0.5 mm. Microscope images are tiled views (3×3) of a single well. An equivalently sized inset for each panel (small white box) is magnified to show plaque detail (upper-right of each panel). See Table 1 for plaque size quantitation and Fig. 2 for plaque size distribution.

**Fig. 2.**
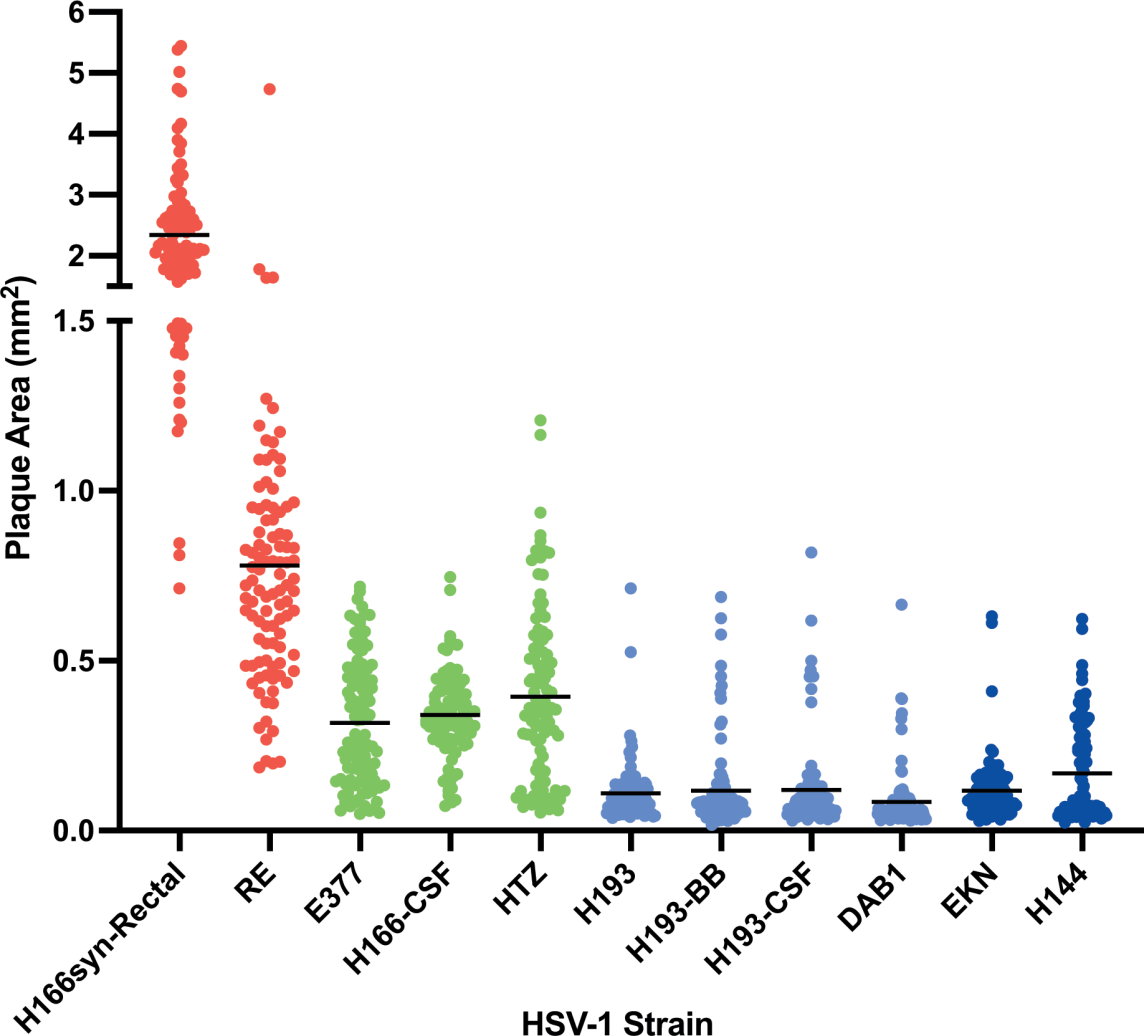
Quantification of plaque sizes for each HSV1 clinical strain reveals large, intermediate and small groupings. Each dot indicates the area of an individual plaque (100 plaques measured for each strain) and horizontal black lines indicate average plaque area. Strains are colour coded according to the groups summarized in Table 1: group 1, red; group 2, green; group 3, light blue; group 4, dark blue.

Eleven clinical isolates of HSV1 were selected for the study based on features of their clinical outcomes, including a range of disease phenotypes in the infected individual and in subsequent quantification of disease severity in a murine model (Table 1). We examined the plaque phenotype of these isolates in cell culture. Evaluation of the strains revealed a range of plaque phenotypes in Vero cells, forming three visually recognizable groups (Fig. 1). Two of the strains formed large plaques that were partially or fully syncytial (Fig. 1a). Three strains were intermediate in their plaque phenotype (Fig. 1b), and six strains produced an average small plaque size (Fig. 1c) (Table 1). We measured 100 plaques per HSV1 strain and found that they differed both in their average plaque size and in their distribution around the mean (Fig. 2).

**Table 1.** Phenotype and origin features of HSV1 strains used in the study

HSV1 strain	Mean plaque area (mm^2^)	Sample collection site*	Human clinical disease*	Virulence in mice*	Group
H166syn	2.34	Rectal	Meningitis and proctitis	13 %	1
RE	0.78	Cornea	Keratitis	50 %	1
E377	0.32	Penis	Genitalis	96 %	2
H166	0.34	CSF	Meningitis and proctitis	87 %	2
HTZ	0.39	Pharynx	Pharyngitis	82 %	2
H193	0.11	Brain	Encephalitis	50 %	3
H193BB	0.12	Brain	Encephalitis	n/a	3
H193CSF	0.12	CSF	Encephalitis	n/a	3
DAB1	0.09	Lip	Labialis	37 %	3
EKN	0.12	Saliva	Gingivostomatitis	87 %	4
H144	0.17	Brain	Encephalitis	86 %	4

*These columns contain previously published data from Dix *et al.* [16].

In a prior study [16], the authors quantified the mortality rate in 4-week-old male BALB/c mice after inoculating 10^6^ p.f.u. of each of the HSV1 isolates into the footpad. Based on these prior results, we classified the HSV1 isolates as either virulent (10^6^ p.f.u. kills >50 % of mice) or attenuated (kills <50 % of mice). This classification, together with plaque size, allowed for discrimination of four groups (Table 1, Fig. 2). Group 1 had large, syncytial plaque growth and an attenuated infection phenotype (strains RE and H166syn-Rectal), while group 2 had an intermediate plaque size and a virulent phenotype in mice (strains H166-CSF, HTZ, E377). Strains with a small average plaque size could be divided into two groups, based on having an attenuated (group 3, strains H193, H193-CSF, H193-BB, DAB1) or a virulent phenotype in mice (Group 4, strains EKN, H144) (Fig. 2).

**Fig. 3.**
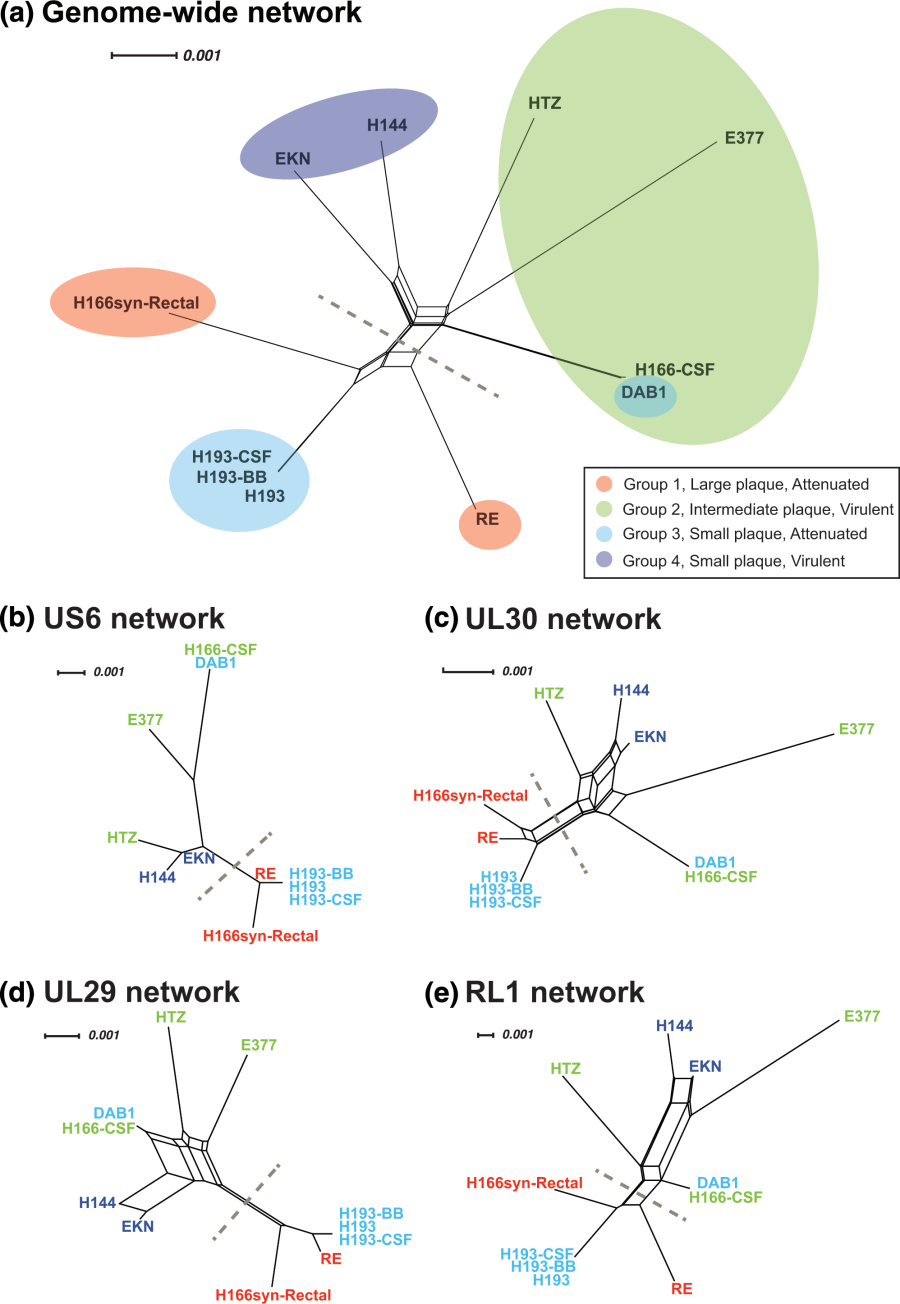
Network graphs of a genome-wide alignment and several gene-specific alignments reveal patterns that appear to separate virulent from attenuated strains. (a) Genome-wide network graph of 11 HSV1 strains showing the genetic relationships between these clinical strains. Grey dashed line indicates the observed separation of attenuated and virulent strains. (b–e) Individual gene network graphs for four of the eight genes (Table 2) that appeared to distinguish the attenuated vs. virulent strains. Alignments and network graphs are based on the DNA sequence of the open reading frame of each gene. Gaps in the alignment were excluded in distance calculations for all trees, and scale bar indicates nucleotide changes per site. Cloud and/or text colours match the group assignment for each strain, as in Fig. 2, where group 1=red; group 2=green; group 3=light blue; group 4=dark blue.

The 11 strains were subjected to whole-genome sequencing and *de novo* assembly (Table S1, available with the online version of this article) to assess their genetic relatedness. We constructed a network graph based on a genome-wide alignment of these isolates (Fig. 3a) and integrated these data with infection and plaque datasets. Results of this integration revealed a separation of attenuated and virulent strains (Fig. 3a dotted line). The attenuated portion of the network graph included group 1, representing two strains with syncytial plaque growth, and group 3, with three strains showing small plaque size. These data suggested genetic similarity among these independent isolates, which share an attenuated disease phenotype in mice (Fig. 3a).

Within the attenuated cluster, three of the strains were collected from a single individual: H193-CSF, H193-BB and H193. These strains were isolated from two body sites – a cerebral spinal fluid isolate (H193-CSF) and a brain biopsy (H193-BB) – and included a later passage of the brain biopsy isolate (H193) [27]. These strains provide an estimate of viral genetic divergence that can arise during host infection and in subsequent cell culture. For the viruses that differ only by passage history, H193-BB and H193, the only differences in coding regions were in the copy number of ‘PQ’ repeats in the gene UL36, which encodes the viral tegument protein VP1/2. Overall these two genomes were 99.61 % identical (excluding the terminal copies of the repeat regions, so that these are not dually represented). Comparison of the coding regions of the isolates from different body sites, H193-BB and H193-CSF, revealed coding differences in the genes UL4 (additional of one proline at AA position 53 in H193CSF) and a repeating tract of mucin-type O-linked glycosylation sites in glycoprotein I, encoded by US7 [28]. Overall these two genomes were 99.86 % identical (excluding the terminal copies of the repeats). These data reflect the potential for genetic drift and/or selective pressures to contribute to genetic change either in or after viral isolation from the host.

In the genomic network graph, two other groups of viral strains (group 2 and 4) appeared to form a virulence cluster (Fig. 3a, above dashed line). One strain within this cluster, H166-CSF, was isolated at the same time as another, H166syn-Rectal, from the same individual. The individual was co-infected with two distinct strains of HSV1, which presented clinically in different body sites [29]. H166syn-Rectal displayed large, nearly uniformly syncytial plaques in culture and an attenuated disease phenotype in mice, whereas H166-CSF had an intermediate (non-syncytial) plaque size in culture and displayed a virulent disease phenotype in mice (Table 1). The strains were also genetically distant as shown in Fig. 3a. These observations indicate that co-occurring HSV1 strains can retain distinct viral features within a single host.

Within the cluster of virulent strains, we also observed an apparent outlier in the viral strain DAB1. DAB1 displayed an attenuated disease phenotype in mice and a small plaque phenotype in culture. The genome sequence of DAB1 demonstrated 99.8 % genetic identity with H166-CSF (excluding terminal copies of the repeats), the virulent strain with intermediate plaque size phenotype described above. Based on this unexpected observation, we prepared new sequencing libraries, resequenced and reassembled these genomes, and re-tested the plaque phenotype using the first-received aliquots of each strain. These data confirmed that DAB1 and H166-CSF were distinct in their plaque phenotypes but extremely similar in genome sequence. This observation implies that a detailed comparison of these two strains may provide important insights into what differentiates virulent and attenuated phenotypes in mice.

Aside from intergenic and repeat regions, the genomes of DAB1 and H166-CSF differed primarily within six genes: US1 (encoding ICP22), US5 (glycoprotein J), UL3, UL24, UL25 (capsid vertex protein) and UL36 (VP1/2). All of these genes except for UL36 revealed sequence differences only in the untranslated regions of each gene, suggesting the potential for differences in gene regulation rather than the direct protein sequences. For UL36, the only differences occurred in the copy number of the PQ amino acid repeat region. Individual gene sequence analysis showed that for each gene region tested, DAB1 and H166-CSF sequences were more similar to each other than to any other strain, suggesting that gene-expression analysis in future studies may supply more informative separation of these two isolates.

To further investigate the separation of attenuated versus virulent strains in the genomic network graph, we tested the ability of individual ORF comparisons to support this attenuated:virulent network discrimination. We examined network graphs for each ORF and found a similar network graph separation with independent sequence comparisons of eight out of 74 ORFs in the HSV1 genome (Table 2). These genes span a range of functions and localizations within the infected cell and/or the virion [30]. This observation suggests that the differential clustering stems, at least in part, from sequence differences in these eight HSV1 genes (Fig. 3b–e; Table 2). While it is not feasible from these data alone to link specific variations in these eight loci with viral attenuation in mice, these genes represent viable candidates for further study.

**Table 2.** Individual HSV1 loci that appear to discriminate between virulent and attenuated clusters

Gene	Gene function*	AA differences†	% AA similarity	Gene differences†	% Gene similarity	Gene length
RL1	Neurovirulence factor ICP34.5	47	81.1 %	90	88.3 %	768 bp
UL21	Viral gene expression / egress tegument protein	9	98.3 %	25	98.4 %	1607 bp
UL29	DNA binding, synthesis	9	99.2 %	62	98.3 %	3591 bp
UL30	DNA polymerase	13	98.9 %	51	98.6 %	3707 bp
UL36	Tegument protein VP1/2	101	96.7 %	214	97.7 %	9222 bp
UL51	Nuclear egress	4	98.4 %	13	98.2 %	735 bp
US6	Envelope glycoprotein D	5	98.7 %	21	98.2 %	1185 bp
US7	Envelope glycoprotein I	22	94.5 %	57	95.2 %	1191 bp

Gene functions from Roizman *et al.* [30]

Differences between the 11 strains listed in Table 1.

**Fig. 4.**
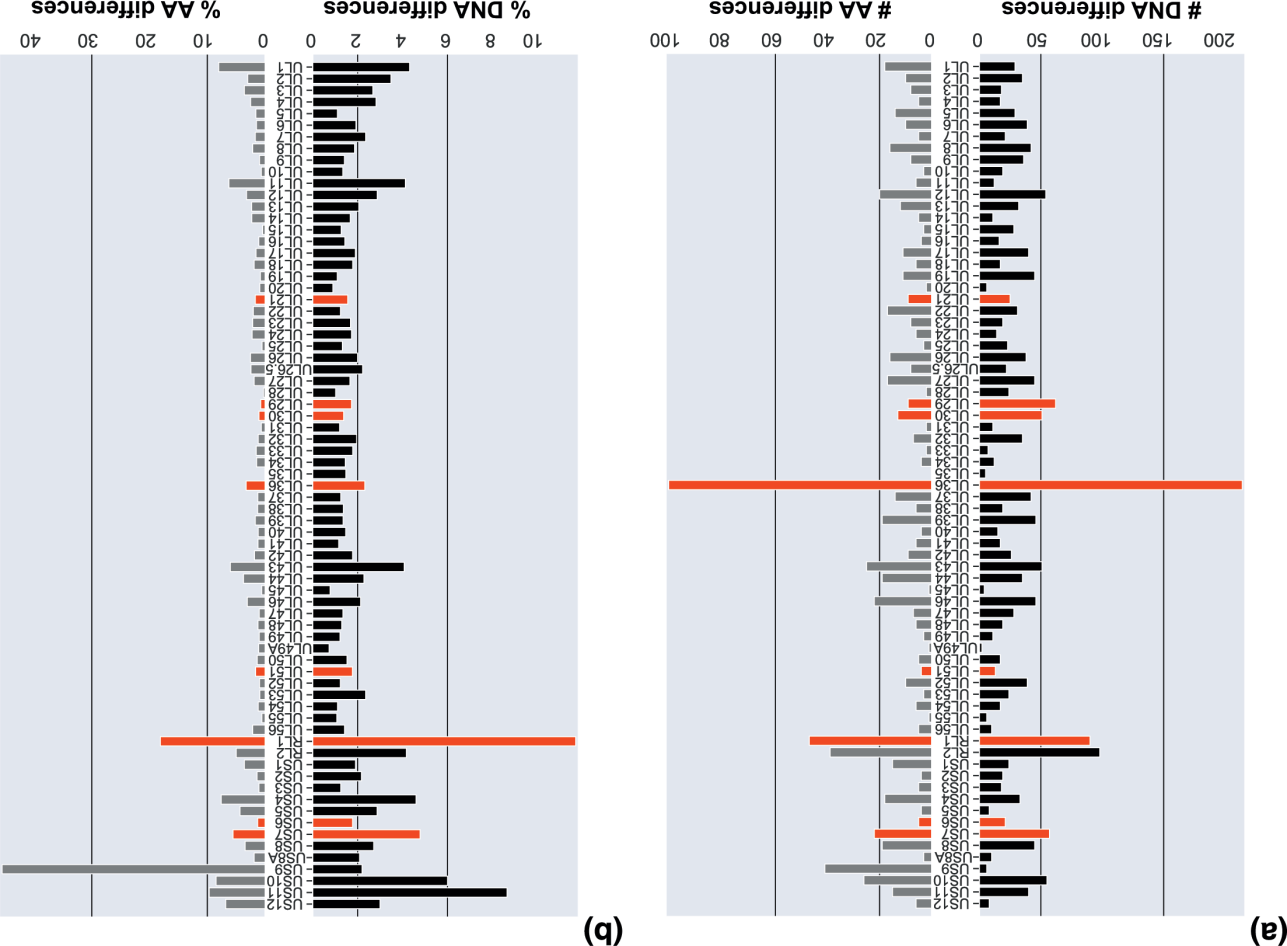
Bar graph quantifying differences in gene and protein variation across 11 HSV1 clinical strains reveal the genome-wide scope of viral genetic diversity. Panel (a) quantifies the number of differences for each gene (left side) and protein (right side), by comparing DNA and amino acid (AA) sequences of each ORF across all 11 clinical strains. Panel (b) quantifies the percent difference (incorporating both the number of differences and the corresponding length) for each gene (left side) and protein (right side). Gene RS1 was excluded due to sequencing gaps and resulting poor alignment. Red bars indicate genes that discriminate attenuated and virulent clusters, as shown in Fig. 3 and Table 2.

Comparative genomic analyses of these 11 strains allowed us to assess the distribution of sequence differences across every viral gene (Fig. 4). We quantified both the raw number of synonymous and non-synonymous differences (Fig. 4a) as well as the percent differences for each ORF and its encoded protein (Fig. 4b). Although we observed variation in the amount of amino acid sequence divergence across the genes, the eight loci that provided discrimination of the attenuated versus virulent phenotypes (Table 2, Fig. 3b–e) were not exceptional in this regard. In the case of RL1, most of the differences in Fig. 4 derive from repeated regions that are challenging to assemble, and where the observed repeat length may not be definitively determined from *de novo* assembly [24, 31]. However, we included this gene in our analysis because the attenuated cluster of the network graph, based on the RL1 ORF (Fig. 3e), remained intact regardless of inclusion or exclusion of gaps in the alignment. Our observations are consistent with the hypothesis that differences between virulent and attenuated phenotypes likely involve subtle and variable changes across multiple genes and/or regulatory regions.

**Fig. 5.**
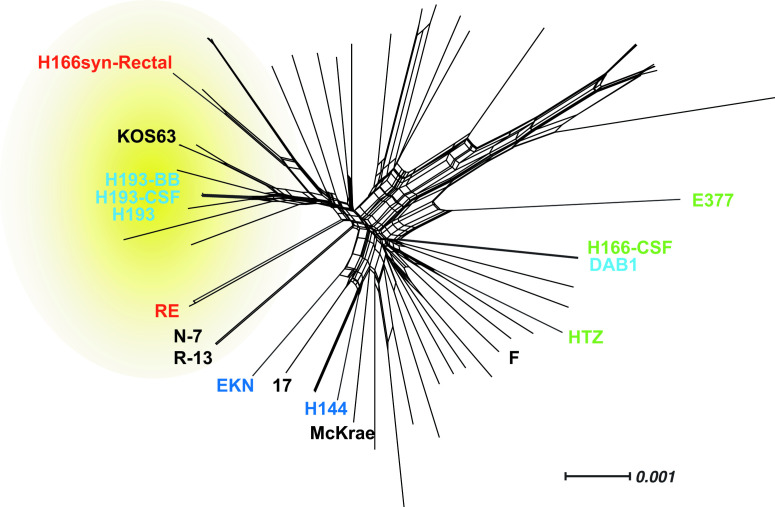
Comparison with a globally representative set of HSV1 strains suggests that these 11 clinical strains encompass a wide array of global diversity. The network graph shown here used a trimmed genome-wide alignment of these 11 clinical isolates with a set of 65 globally representative comparison strains (Table S2). The yellow cloud indicates the area of the graph containing several strains of attenuated virulence in mice. Gaps in the alignment were excluded in distance calculations, and the scale bar indicates the number of nucleotide differences per site. Strain name colours correspond to the groups shown in Figs. 2–3 and Table 1. Well-known HSV1 strains with previously characterized murine virulence levels are labelled in black for comparison. HSV1 strains KOS63 and the father–son pair of R-13 and N-7 have been previously characterized to have an attenuated virulence phenotype *in vivo* in murine models of infection [16, 32–34]. In contrast, HSV1 strains McKrae, 17 and F display higher levels of virulence *in vivo* [16, 33, 35, 36]. The full list of strains included in the network graph can be found in Table S2.

We examined relatedness of these 11 HSV1 isolates to a collection of HSV1 strains representing the overall global diversity of the pathogen (Table S2). The 11 isolates were relatively well-distributed in the network graph, indicating a diverse set of samples. Within the larger population of global HSV1 strains, we observed evidence of sequence clustering between the attenuated strains from this study and several strains from the larger group (Fig. 5). Within this portion of the network graph, HSV1 strains KOS63 and the father–son pair R-13 and N-7 have been previously shown to display an attenuated infection phenotype [16, 32–34]. Outside of this potential attenuated cluster, HSV1 strains McKrae, 17 and F have been previously shown to display higher levels of virulence *in vivo* [16, 33, 35, 36]. This observation raises the intriguing possibility that other strains in the same region as the attenuated cluster of the network graph may be attenuated in mice, although the *in vivo* virulence phenotypes of these strains are not yet known.

## Discussion

The analysis of clinical strains provides an important value for understanding the genetic diversity and phenotypic impacts of HSV1 infection and disease. Clinical strains offer insight into viral phenotypes that cannot be adequately captured in studies that are limited to lab-adapted strains. Here, we present a detailed characterization of 11 HSV1 clinical strains that reveal several unusual observations. For example, two strains in this collection were derived from a single patient who had coincident infections with two genetically and phenotypically distinct strains (H166-CSF and H166syn-Rectal [29]). This individual presents a rare example of distinct, dual-strain infection. In most cases, viruses sampled from the same individual have been found to share an overall similar consensus genome [37–40], as we observed with the H193 isolates [27]. Conversely, two strains in this collection that were derived from different individuals were found to have distinct murine infection and plaque phenotypes but with nearly identical genomes (DAB1 and H166-CSF). These types of observations would likely have gone unnoticed without the integration of multiple forms of phenotypic and genomic data. This study underlines the value of multifaceted approaches to advance our understanding of viral diversity.

The incorporation of plaque phenotype into studies of viral genomes and/or animal models of virulence is relatively unusual [24, 41]. However, it was the inclusion of plaque size quantitation that permitted discrimination of DAB1 from its near-identical genetic neighbour H166-CSF, and it provided additional discrimination within the virulence cluster of the network graph. As cell- and animal-based phenotype information accumulates for greater numbers of viral isolates, it is likely that more comprehensive data will provide greater power to model the mechanisms of virulence vs. attenuation [7, 16, 20, 21, 32, 33]. Thus far, we and others have found Vero cells to be the most useful cell line for its ability to discriminate HSV strains based on plaque phenotype and size [24, 41–44]. However these cells are both non-human (African green monkey) and defective in interferon-signalling [45, 46]. Further studies will be needed to explore how plaque phenotypes manifest in human cells with fully functional interferon pathways.

The genetic relationship identified for H166-CSF and DAB1 presents an uncommon opportunity to identify genomic determinants of virulence. Given the high degree of relatedness between the coding sequences of these genomes, it is possible that noncoding regions of the genome are influencing virulence. Despite a substantial body of literature on HSV1 transcriptional and translational mechanisms, we still lack data on how sequence differences in these noncoding and/or regulatory regions impact HSV1 gene expression and protein turnover [47–55]. Future studies could utilize high-resolution gene expression time course data to deduce how subtle genetic differences lead to large phenotype changes [47], e.g. through transcriptional or translational regulatory mechanisms. We anticipate that the combination of plaque phenotype data, together with gene expression features, will help to decipher how DAB1 diverged in phenotype and virulence from its near-identical genetic neighbour, H166-CSF.

For the present categorization of HSV1 strains, we used three datasets that compared plaque size, viral genome sequence and murine disease phenotype after peripheral infection. We did not include the original clinical diagnoses in this categorization, although the information is reported in Table 1. The reason for this decision is that human clinical outcomes are impacted by many factors, including host age, route and dose of infection, and co-morbid factors [56–59]. Prior studies have also shown that differences in host immunity impact human responses to HSV1 infection [60–63]. The value of a murine model of infection for phenotypic comparisons of virulence is that it allows for the standardization of host immunity, infectious dose and route of viral administration. Even within the mouse infection data, outcomes differed between footpad and intracerebral routes of infection and the inoculation dose used [16]. We were able to distinguish an attenuated cluster in the network graph of these HSV1 strains using integrated genetic, murine and plaque data. We focused on the murine footpad route of infection for virulence assessment because it better mimicks peripheral sites of HSV1 introduction in human patients. We found that incorporation of mouse intracerebral infection data offered no meaningful discrimination value in this analysis, due to the high mortality observed in mice with this unnatural route of infection. These observations support the value of using a standardized murine model to quantitatively measure the virulence of HSV1 strains, in combination with additional phenotypic and genetic measures.

An important outcome of this study was the detection of an attenuated cluster of strains within the initial 11-strain analysis. This cluster enabled the identification of a larger potential cluster of attenuated strains when the data were combined into a larger HSV1 network graph. We consider this observation to be an important outcome of an integrative analysis approach. The same approach of incorporating plaque size with genomic and infection features should allow us to discriminate additional aspects of the genetic clustering of virulent strains as well, and to begin to explore related mechanisms of virulence.

## Supplementary Data

Supplementary material 1
