# Supplementary material for: Multi-phenotype analysis for enhanced classification of 11 herpes simplex virus 1 strains
Source: J Gen Virol. 2022 Oct 19;103(10):001780. doi: 10.1099/jgv.0.001780 (PMC10019087; doi:10.1099/jgv.0.001780)
Supplement: Supplementary material 1 [file jgv-103-1780-s001.pdf]

Table S1. Sequencing statistics for 11 HSV1 strains

Sample Name	Average coverage	No. of raw sequencing reads	No. of reads used for assembly	GenBank accession number	Additional reference*
**H166syn-Rectal**	8,443	5.7 million	3.1 million	KM222727	(23)
**H166-CSF**	23,258	10.5 million	9.9 million	KM222726	(23)
**H193**	17,661	7.6 million	7.3 million	KT425108	(24)
**H193-CSF**	5,072	5.5 million	5.3 million	ON960054	(24)
**H193-BB**	3,743	5.1 million	4.9 million	ON960055	(24)
**RE**	4,657	6.3 million	5.9 million	ON960060	(25)
**HTZ**	4,668	5.8 million	5.2 million	ON960059	(26)
**EKN**	3,085	4.7 million	2.5 million	ON960056	(5)
**E377**	6,643	5.5 million	5.3 million	ON960061	(5)
**DAB1**	9,963	6.1 million	4.8 million	ON960057	(5)
**H144**	6,018	5.5 million	2.4 million	ON960058	(5)

* All of these strains were also characterized *in vivo* in mice in Dix et al. (5)

Table S2: GenBank accession numbers and references for previously sequenced isolates used in network graph analysis.

Virus	Strain Origin	GenBank Accession	Refs.*
17	Glasgow, UK	JN555585 NC_001806	(1, 2)
F	Chicago, IL	GU734771	(3, 4)
H129	San Francisco, CA	GU734772	(3, 5)
KOS	Houston, TX	JQ673480, JQ780693	(6, 7)
McKrae	Gainesville, FL	JQ730035, JX142173	(8–10)
HF10	New York, NY	DQ889502	(11)
KOS63	Houston, TX	KT425110	(12)
KOS79	Houston, TX	KT425109	(12)
India	Pune, India	KJ847330	(13)
L2	Moscow, Russia	KT780616	(14)
SC16	Madrid, Spain	KX946970	(15)
MacIntyre	Berkeley, CA	KM222720	(16)
CJ970	Madison, WI	JN420341.1	(17)
CJ311	Madison, WI	JN420338.1	(17)
134	Madison, WI	JN4000093.1	(18)

Virus	Strain Origin	GenBank Accession	Refs.*
RE	New Orleans, LA	KF498959	N/A
160/1982	Erfurt, Germany	LT594192	(19)
132/1998	Gelsenkirchen, Germany	LT594457	(19)
1394/2005	Germany	LT594111	(19)
1319/2005	Germany	LT594108	(19)
66/2007	Jena, Germany	LT594110	(19)
369/2007	Jena, Germany	LT594112	(19)
3083/2008	Jena, Germany	LT594107	(19)
270/2007	Manebach, Germany	LT594109	(19)
2158/2007	Jena, Germany	LT594106	(19)
172/2010	Jena, Germany	LT594105	(19)
CR38	Shenyang, China	HM585508	(20)
E07	Nairobi, Kenya	HM585497	(20)
E06	Nairobi, Kenya	HM585496	(20)
E08	Nairobi, Kenya	HM585498	(20)

Virus	Strain Origin	GenBank Accession	Refs.*
E10	Nairobi, Kenya	HM585499	(20)
E11	Nairobi, Kenya	HM585500	(20)
E12	Nairobi, Kenya	HM585501	(20)
E13	Nairobi, Kenya	HM585502	(20)
E14	Nairobi, Kenya	HM585510	(20)
E15	Nairobi, Kenya	HM585503	(20)
E19	Nairobi, Kenya	HM585511	(20)
E22	Nairobi, Kenya	HM585504	(20)
E23	Nairobi, Kenya	HM585505	(20)
E25	Nairobi, Kenya	HM585506	(20)

Virus	Strain Origin	GenBank Accession	Refs.*
E35	Nairobi, Kenya	HM585507	(20)
R11	Seoul, South Korea	HM585514	(20)
R62	Seoul, South Korea	HM585515	(20)
S23	Sapporo, Japan	HM585512	(20)
S25	Sapporo, Japan	HM585513	(20)
N-7	Cincinnati, OH	KY922719	(21)
R-13	Cincinnati, OH	KY922718	(21)
v.29	Seattle, WA	MH102298	(22)

References

1. McGeoch DJ, Dalrymple MA, Davison AJ, Dolan A, Frame MC, McNab D, Perry LJ, Scott JE, Taylor P. 1988. The complete DNA sequence of the long unique region in the genome of herpes simplex virus type 1. J Gen Virol 69:1531–74.
2. McGeoch DJ, Dolan A, Donald S, Rixon FJ. 1985. Sequence determination and genetic content of the short unique region in the genome of herpes simplex virus type 1. J Mol Biol 181:1–13.
3. Szpara ML, Parsons L, Enquist LW. 2010. Sequence variability in clinical and laboratory isolates of herpes simplex virus 1 reveals new mutations. J Virol 84:5303–13.
4. Ejercito PM, Kieff ED, Roizman B. 1968. Characterization of herpes simplex virus strains differing in their effects on social behaviour of infected cells. J Gen Virol 2:357–364.
5. Dix RD, McKendall RR, Baringer JR. 1983. Comparative neurovirulence of herpes simplex virus type 1 strains after peripheral or intracerebral inoculation of BALB/c mice. Infect Immun 40:103–112.
6. Macdonald SJ, Mostafa HH, Morrison LA, Davido DJ. 2012. Genome sequence of herpes simplex virus 1 strain KOS. J Virol 86:6371–6372.
7. Smith KO. 1964. Relationship Between the Envelope and the Infectivity of Herpes Simplex Virus. Exp Biol Med 115:814–816.
8. Watson G, Xu W, Reed A, Babra B, Putman T, Wick E, Wechsler SL, Rohrmann GF, Jin L. 2012. Sequence and comparative analysis of the genome of HSV-1 strain McKrae. Virology 433:528–37.
9. Macdonald SJ, Mostafa HH, Morrison LA, Davido DJ. 2012. Genome sequence of herpes simplex virus 1 strain McKrae. J Virol 86:9540–9541.
10. Williams LE, Nesburn AB, Kaufman HE. 1965. Experimental induction of disciform keratitis. Arch Ophthalmol 73:112–114.
11. Ushijima Y, Luo C, Goshima F, Yamauchi Y, Kimura H, Nishiyama Y. 2007. Determination and analysis of the DNA sequence of highly attenuated herpes simplex virus type 1 mutant HF10, a potential oncolytic virus. Microbes Infect 9:142–149.

12. Bowen CD, Renner DW, Shreve JT, Tafuri Y, Payne KM, Dix RD, Kinchington PR, Gatherer D, Szpara ML. 2016. Viral forensic genomics reveals the relatedness of classic herpes simplex virus strains KOS, KOS63, and KOS79. Virology 492:179–186.

13. Bondre VP, Sankararaman V, Andhare V, Tupekar M, Sapkal GN. 2016. Genetic characterization of human herpesvirus type 1: Full-length genome sequence of strain obtained from an encephalitis case from India. Indian J Med Res 144:750–760.

14. Skoblov MYu, Lavrov AV, Bragin AG, Zubtsov DA, Andronova VL, Galegov GA, Skoblov YuS. 2017. The genome nucleotide sequence of herpes simplex virus 1 strain L2. Russ J Bioorganic Chem 43:140–142.

15. Rastrojo A, López-Muñoz AD, Alcamí A. 2017. Genome Sequence of Herpes Simplex Virus 1 Strain SC16. Genome Announc 5:e01392-16.

16. Szpara ML, Tafuri YR, Parsons L, Shreve JT, Engel EA, Enquist LW. 2014. Genome sequence of the anterograde-spread-defective herpes simplex virus 1 strain MacIntyre. Genome Announc 2.

17. Kolb AW, Adams M, Cabot EL, Craven M, Brandt CR. 2011. Multiplex Sequencing of Seven Ocular Herpes Simplex Virus Type-1 Genomes: Phylogeny, Sequence Variability, and SNP Distribution. Investig Opthalmology Vis Sci 52:9061.

18. Kolb AW, Lee K, Larsen I, Craven M, Brandt CR. 2016. Quantitative Trait Locus Based Virulence Determinant Mapping of the HSV-1 Genome in Murine Ocular Infection: Genes Involved in Viral Regulatory and Innate Immune Networks Contribute to Virulence. PLOS Pathog 12:e1005499.

19. Pfaff F, Groth M, Sauerbrei A, Zell R. 2016. Genotyping of herpes simplex virus type 1 (HSV-1) by whole genome sequencing. J Gen Virol https://doi.org/10.1099/jgv.0.000589.

20. Szpara ML, Gatherer D, Ochoa A, Greenbaum B, Dolan A, Bowden RJ, Enquist LW, Legendre M, Davison AJ. 2014. Evolution and diversity in human herpes simplex virus genomes. J Virol 88:1209–27.

21. Pandey U, Renner DW, Thompson RL, Szpara ML, Sawtell NM. 2017. Inferred father-to-son transmission of herpes simplex virus results in near-perfect preservation of viral genome identity and in vivo phenotypes. Sci Rep 7:13666.

22. Shipley MM, Renner DW, Ott M, Bloom DC, Koelle DM, Johnston C, Szpara ML. 2018. Genome-wide surveillance of genital herpes simplex virus type 1 from multiple anatomic sites over time. J Infect Dis 218:595–605.

23. Heller M, Dix RD, Baringer JR, Schachter J, Conte JE Jr. 1982. Herpetic proctitis and meningitis: recovery of two strains of herpes simplex virus type 1 from cerebrospinal fluid. J Infect Dis 146:584–588.

24. Dix RD, Lukes S, Pulliam L, Baringer JR. 1983. DNA restriciton Enzyme Analysis of Viruses Isolated From Cerebrospinal Fluid and Brain-Biopsy Tissue in a Patient with herpes Simplex Encephalitis. N Engl J Med 1424.

25. Irvine AR, Kimura SJ. 1967. Experimental Stromal Herpes Simplex Keratitis in Rabbits. Arch Ophthalmol 78:654–663.

26. McKendall RR, Vogelzang N, Jackson GG. 1974. Herpes Virus Latency in Spinal Ganglia of Mice Without Illness. Exp Biol Med 146:1093–1096.
